# Changes in failure to rescue after gastrectomy at a large-volume center with a 16-year experience in Korea

**DOI:** 10.1038/s41598-023-32593-6

**Published:** 2023-03-31

**Authors:** Sung Hyun Park, Ki-Yoon Kim, Minah Cho, Yoo Min Kim, Woo Jin Hyung, Hyoung-Il Kim

**Affiliations:** 1 Department of Surgery, Yonsei University College of Medicine, 50-1 Yonsei-ro Seodaemun-gu, Seoul, 03722 Republic of Korea; 2 Gastric Cancer Center, Yonsei Cancer Center, Yonsei University Health System, Seoul, South Korea

**Keywords:** Gastric cancer, Gastric cancer

## Abstract

Failure to rescue (FTR), the mortality rate among patients with complications, is gaining attention as a hospital quality indicator. However, comprehensive investigation into FTR has rarely been conducted after radical gastrectomy for gastric cancer patients. This study aimed to assess FTR after radical gastrectomy and investigate the associations between FTR and clinicopathologic factors, operative features, and complication types. From 2006 to 2021, 16,851 gastric cancer patients who underwent gastrectomy were retrospectively analyzed. The incidence and risk factors were analyzed for complications, mortality, and FTR. Seventy-six patients had postoperative mortality among 15,984 patients after exclusion. The overall morbidity rate was 10.49% (1676/15,984 = 10.49%), and the FTR rate was 4.53% (76/1676). Risk factor analysis revealed that older age (reference: < 60; vs. 60–79, adjusted odds ratio [OR] 2.07, 95% confidence interval [CI] 1.13–3.79, *P* = 0.019; vs. ≥ 80, OR 3.74, 95% CI 1.57–8.91, *P* = 0*.*003), high ASA score (vs. 1 or 2, OR 2.79, 95% CI 1.59–4.91, *P* < 0.001), and serosa exposure in pathologic T stage (vs. T1, OR 2.74, 95% CI 1.51–4.97, *P* < 0.001) were associated with FTR. Moreover, patients who underwent gastrectomy during 2016–2021 were less likely to die when complications occurred than patients who received the surgery in 2006–2010 (OR 0.35, 95% CI 0.18–0.68, *P* = 0.002). This investigation of FTR after gastrectomy demonstrated that the risk factors for FTR were old age, high ASA score, serosa exposure, and operation period. FTR varied according to the complication types and the period, even in the same institution.

## Introduction

Gastric cancer is the fifth most commonly diagnosed cancer and the third leading cause of cancer-related death globally^1^. Surgical resection is the primary treatment for gastric cancer and provides the best opportunity to cure these patients. Although surgeons are making substantial efforts to reduce postoperative complications, morbidity rates of up to 10–28% have been reported^2–7^. Due to the technical demands of anastomosis and D2 lymphadenectomy, radical gastrectomy still has procedural complexities. Surgeons should always consider the possibility of fatal complications, such as anastomosis leakage, pancreatic fistula, and splenic artery aneurysm.

It is essential to reduce morbidity after surgery and to decrease mortality with appropriate management when complications develop. Failure to rescue (FTR) is the mortality rate among patients with postoperative complications and is gaining attention as an index for the proper management of postoperative complications^8–11^. Early detection of complications followed by immediate and effective interventions would help patients avoid lethal progression of the disease. Furthermore, understanding the factors associated with FTR may enable surgeons to avoid mortality.

To the best of our best knowledge, no large-scale comprehensive study has analyzed FTR after radical gastrectomy in gastric cancer patients, particularly in the East Asia region where gastric cancer is prevalent^7,12,13^. Moreover, no analysis has examined FTR variation by operation period, which may represent the changes in the hospital system and the development of surgical technology. This comprehensive analysis of FTR in a high-volume center from 2006 to 2021 aimed to investigate the risk factors of FTR and the treatment outcomes by type of complication.

## Methods

### Patients

We reviewed a prospectively collected gastric cancer database, including 16,851 patients who underwent gastrectomy for gastric cancer from January 2006 to December 2021 at Severance Hospital (Seoul, Republic of Korea). Patient and tumor characteristics, surgical features, and pathologic information were collected. Depending on the location and clinical stage of the lesion, subtotal gastrectomy, total gastrectomy, or proximal gastrectomy was conducted. According to the Korean Practice Guideline for Gastric Cancer, D1 + lymphadenectomy was performed for early gastric cancer patients with any suspicion of LN metastasis, and D2 lymphadenectomy was performed for patients with suspicion of LN metastasis or advanced gastric cancer^14^. The exclusion criteria were preoperative palliative-aimed chemotherapy or radiation therapy for current gastric cancer, M1 patients, R1 or R2 resection, and incomplete information on clinical or pathological features. This study was approved by the Institutional Review Board of Severance Hospital, Yonsei University Health System (2022-2370-001), which waived informed consent for the study because of its retrospective nature. All methods were performed in accordance with the relevant guidelines and regulations.

### Complication and mortality data collection

The primary outcomes were morbidity, mortality, and FTR, as defined in Table 1^15^. We collected details on major complications and readmission. To confirm that the data collection was sound, retrospectively collected data were compared with prospectively collected data. In our institution, we collected any postoperative complications and readmissions weekly from January 2013 for quality control. Using those data as ground truth, we developed a systematic workflow to retrospectively detect complications. After confirming the similar quality of the retrospective and prospective data, the data capture was extended to January 2006. We reviewed the medical records from the entire hospitalization or readmission period of the patients who underwent gastrectomy between January 2006 and December 2012 to determine which complications occurred. Both the medical records and survey data from the National Statistical Office of Korea were used to verify patient mortality.

**Table 1:** Definitions of morbidity, mortality, failure to rescue (FTR), and index complication with a list of complication types.

Terms	Definitions
Morbidity	Grade III or higher complication based on Clavien-Dindo classification, or patient required re-hospitalization within 90 days after surgery due to postoperative complications^15^
Mortality	Death of the patient due to postoperative complication within 90 days or during the hospitalization period
Failure-to-rescue rate	Mortality among patients with morbidity
Causal complication	Complication that led to subsequent complication (e.g., anastomosis leakage and intraabdominal abscess)
Index complication	Complications that had the most significant influence on the patient’s postoperative course

Complication type	
Surgical complication	Systemic complication
Anastomosis leakage	Cardiac complication
Esophagojejunostomy leakage	Coronary artery disease
Duodenal stump leakage	Arrhythmia
Other anastomosis leakage	Other cardiac complication
Anastomosis stenosis	Pulmonary complication
Postoperative bleeding	Pleural effusion
Intraabdominal bleeding	Pneumonia
Intraluminal bleeding	Acute respiratory distress syndrome
Pancreas-related complication	Desaturation
Postoperative fluid collection	Other pulmonary complication
Sterile fluid collection	Renal complication
Chylous ascites	Urologic complication
Intraabdominal abscess	Cerebrovascular disease
Intestinal obstruction	Other medical complication
Gastrostasis or ileus	
Other surgical complication	

### Postoperative complications

We divided postoperative complications into surgical and systemic complications and reviewed each patient for details regarding the kind of complications they developed (Table 1). If the causal relationship between multiple complications was clear (e.g., cause: anastomosis leakage, result: intraabdominal abscess), a preceding complication was classified as a causal complication. If multiple complications occurred in the same patient, the index complication was defined as the complication with the highest grade or the most critical complication for the patient’s outcome if the grades were the same.

### Statistical analysis

Statistical analysis was performed using R (Version 4.2.0; R Foundation for Statistical Computing, Vienna, Austria). The following variables were used in the analysis of morbidity, mortality, and FTR rate: patient demographics (age, sex, body mass index [BMI], and American Society of Anesthesiology [ASA] score), operation period, surgical extent (resection extent and lymph node dissection extent), and pathologic tumor stage (pathologic T stage and N stage)." Continuous variables were described as mean (standard deviation) or median (interquartile range) depending on whether the variable had a normal distribution. Categorical variables were described as numbers (percentages). Groups were compared using the Student’s *t*-test or Mann–Whitney U test for continuous variables and the Chi-square or Fisher’s exact test for categorical variables. The multivariable logistic regression model was used to demonstrate the risk factors for morbidity, mortality, and FTR. Variable selection for this model was performed using a stepwise method, which enables the selection of the most suitable variables for logistic regression. *P-*values less than 0.05 were considered significant.

## Results

### Patients

**Figure 1:**
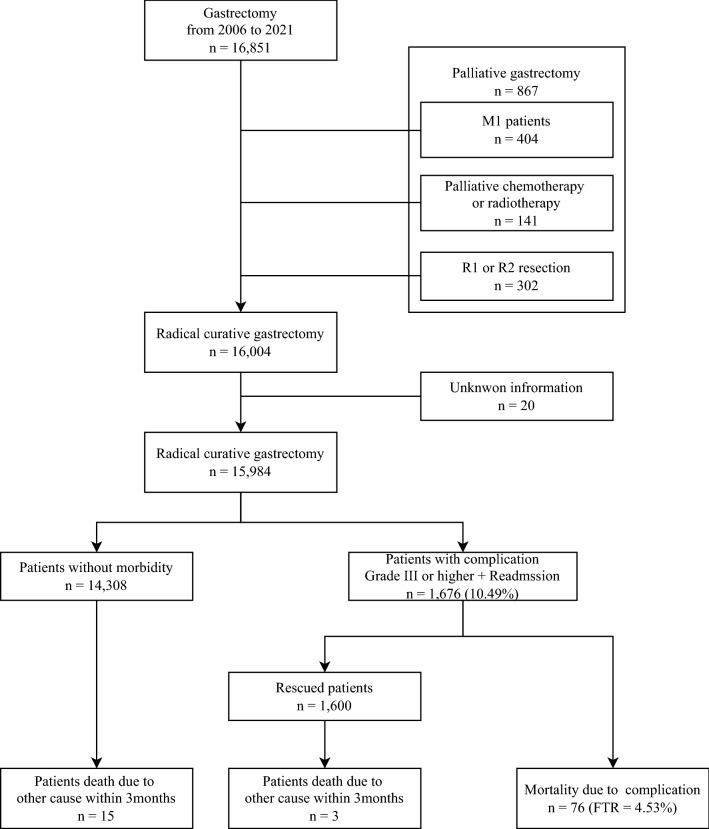
Study profile.

From January 2006 to December 2021, 16,851 patients underwent gastrectomy for gastric cancer in our institution. Among them, 404 patients were preoperative stage M1 and 141 patients were excluded because they received preoperative palliative chemotherapy. We also excluded 302 patients who received R1 or R2 resection, not curative R0 resection, and 20 patients with incomplete clinicopathologic data, ultimately including 15,984 patients in our analysis (Fig. 1). Overall morbidity occurred in 1676 patients (1676/15,984, 10.49%) who developed grade III or higher complications or required readmission due to postoperative complications. Among them, mortality due to postoperative complications occurred in 76 patients (76/15,984, 0.48%), and the FTR rate was 4.53% (76/1676).

### Characteristics of patients with complications

Supplementary Table S1 presents a comparison of the characteristics, surgical factors, and pathologic features of 1,676 morbidity patients and 14,308 patients without morbidity. There were significant differences in age (*P* < 0.001), sex (*P* < 0.001), ASA score (*P* < 0.001), operation method (*P* < 0.001), resection extent (*P* < 0.001), dissection extent (*P* < 0.001), and pathologic stage (pT, *P* < 0.001; pN, *P* < 0.001) between patients with and without complications. There was no difference in the morbidity rate according to the year of operation (*P* = 0.714). The morbidity rate was 10.80% from 2006 to 2010, 10.31% from 2011 to 2015, and 10.39% from 2016 to 2021.

### Characteristics of FTR patients

Supplementary Table S2 presents a comparison of 76 mortality patients whose complications led to death and 1,600 rescued patients who recovered. Rescued patients and those who experienced mortality differed in age (*P* < 0.001), BMI group (*P* = 0.019), ASA score (*P* = 0.001), dissection extent (*P* = 0.033), and pathologic stage (pT, *P* = 0.005; pN, *P* = 0.034). Once complications happened, there were no differences in sex (*P* = 0.597), operation method (*P* = 0.966), or resection extent (*P* = 0.714).

### FTR according to clinicopathological features and surgical factors

**Figure 2:**
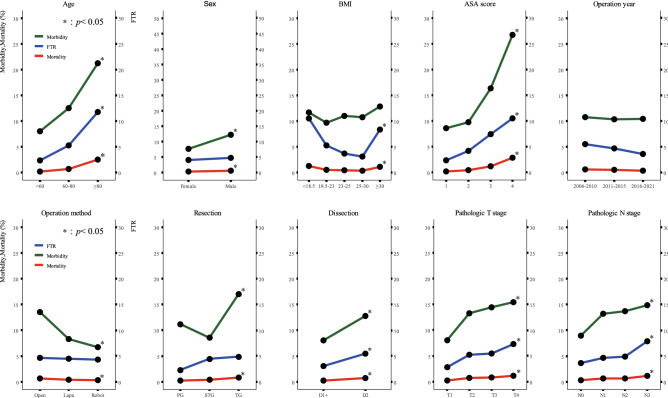
Morbidity, mortality, and failure to rescue (FTR) according to clinicopathologic and surgical factor (groups were compared using the Chi-square test or Fisher’s exact test. *P < 0.05. Green and red lines represent morbidity and mortality rates, respectively, and are displayed on the left axis. The blue line indicates the FTR rate and is displayed on the right y-axis.)

Figure 2 shows morbidity, mortality, and FTR according to clinicopathologic features and surgical factors. Age, ASA score, dissection extent, and pathologic stage were related to both the development of morbidity and FTR. Sex, operation method, and resection extent affected morbidity but not FTR. Morbidity was similar among BMI groups, but the FTR rate was higher for patients with malnutrition (BMI < 18.5 kg/m^2^) and obese patients (BMI ≥ 30 kg/m^2^).

### Multivariable logistic regression model for risk factor analysis of morbidity, mortality, and FTR

Table 2 shows the univariable and multivariable logistic regression results for morbidity, mortality, and FTR risk factor analysis. Older age, male sex, high ASA score, open surgery, total gastrectomy or proximal gastrectomy, and advanced pathologic T stage were risk factors for the development of morbidity. Risk factor analysis also revealed that older age (reference: < 60; vs. 60–79, adjusted odds ratio [OR] 2.07, 95% confidence interval [CI] 1.13–3.79, *P* = 0.019; vs. ≥ 80, OR 3.74, 95% CI 1.57–8.91, *P* = 0.003), high ASA score (vs. 1 or 2, OR 2.79, 95% CI 1.59–4.91, *P* < 0.001), and serosa exposure in pathologic T stage (vs. T1, OR 2.74 95% CI 1.51–4.97, *P* < 0.001) were associated with FTR. Furthermore, patients who underwent gastrectomy in 2016–2021 were less likely to die when complications occurred than patients who received the surgery in 2006–2010 (OR 0.35, 95% CI 0.18–0.68, *P* = 0.002). Subgroup analysis for leakage (N = 163, Supplementary Table S3), one of the most critical complications, showed a decreased FTR rate in the more recent period (2016–2021 vs. 2006–2010, OR 0.14, 95% CI 0.03–0.76, *P* = 0.023) in the multivariable logistic regression analysis.

**Table 2:** Logistic regression models for risk factor analysis of morbidity, mortality, and failure to rescue after gastrectomy.

Variables	Comparison	Morbidity				Mortality				Failure to rescue			
		Uni		Multi		Uni		Multi		Uni		Multi	
		OR (95% CI)	*P-*value	OR (95% CI)	*P-*value	OR (95% CI)	*P-*value		*P-*value	OR (95% CI)	*P-*value	OR (95% CI)	*P-*value
Age, yr (ref: < 60)	vs. 60–79	1.65 (1.48–1.83)	< 0.001	1.42 (1.27–1.58)	< 0.001	3.48 (1.95–6.20)	< 0.001	2.58 (1.42–4.69)	0.002	2.28 (1.27–4.09)	0.006	2.07 (1.13–3.79)	0.019
	vs. ≥ 80	3.11 (2.44–3.96)	< 0.001	2.49 (1.92–3.23)	< 0.001	13.42 (6.13–29.38)	< 0.001	6.49 (2.76–15.22)	< 0.001	5.44 (2.42–12.25)	< 0.001	3.74 (1.57–8.91)	0.003
Sex (ref: female)	vs. Male	1.67 (1.31–1.65)	< 0.001	1.47 (1.31–1.65)	< 0.001	1.89 (1.11–3.23)	0.018	1.61 (0.94–2.78)	0.080	1.20 (0.70–2.04)	0.51	NA	
BMI, kg/m^2^ (ref: 18.5–22.9)	vs. < 18.5	1.23 (0.96–1.59)	0.104	NA		2.43 (1.12–5.29)	0.025	NA		2.12 (0.94–4.78)	0.070	NA	
	vs. 23–24.9	1.15 (1.02–1.31)	0.028	NA		0.79 (0.44–1.42)	0.424	NA		0.68 (0.38–1.24)	0.211	NA	
	vs. 25–29.9	1.13 (0.99–1.28)	0.067	NA		0.65 (0.35–1.21)	0.172	NA		0.57 (0.30–1.08)	0.082	NA	
	vs. ≥ 30	1.37 (1.00–1.88)	0.048	NA		2.11 (0.74–5.99)	0.160	NA		1.64 (0.56–4.84)	0.371	NA	
ASA score (ref: 1,2)	vs. 3, 4	1.99 (1.77–2.24)	< 0.001	1.79 (1.57–2.04)	< 0.001	4.18 (2.65–6.58)	< 0.001	3.99 (2.28–6.97)	< 0.001	2.37 (1.49–3.78)	< 0.001	2.79 (1.59–4.91)	< 0.001
Op period (ref: 2006–2010)	vs. 2011–2015	0.95 (0.84–1.08)	0.444	NA		0.82 (0.48–1.40)	0.458	0.59 (0.33–1.05)	0.072	0.85 (0.49–1.47)	0.553	0.62 (0.34–1.11)	0.107
	vs. 2016–2021	0.96 (0.85–1.09)	0.515	NA		0.64 (0.37–1.11)	0.111	0.32 (0.17–0.62)	< 0.001	0.65 (0.37–1.11)	0.135	0.35 (0.18–0.68)	0.002
Op method (ref: Open)	vs. Lapa	0.58 (0.52–0.65)	< 0.001	0.62 (0.55–0.70)	< 0.001	0.59 (0.35–0.98)	0.042	NA		0.95 (0.56–1.61)	0.860	NA	
	vs. Robot	0.46 (0.39–0.54)	< 0.001	0.55 (0.46–0.65)	< 0.001	0.45 (0.21–0.96)	0.039	NA		0.91 (0.43–1.97)	0.819	NA	
Resection (ref: STG)	vs. TG	2.19 (1.96–2.44)	< 0.001	1.83 (1.63–2.06)	< 0.001	2.18 (1.36–3.47)	0.001	NA		1.09 (0.68–1.76)	0.710	NA	
	vs. PG	1.34 (0.97–1.85)	0.071	1.70 (1.22–2.37)	0.002	0.67 (0.09–4.48)	0.687	NA		0.50 (0.07–3.71)	0.497	NA	
Dissection (ref: D1 +)	vs D2	1.67 (1.51–1.85)	< 0.001	NA		2.83 (1.68–4.77)	< 0.001	NA		1.82 (1.07–3.10)	0.026	NA	
pT stage (ref: T1)	vs. T2	1.75 (1.49–2.04)	< 0.001	1.38 (1.17–1.62)	< 0.001	3.09 (1.53–6.26)	0.002	2.5 (1.23–5.08)	0.012	1.92 (0.93–3.93)	0.076	1.77 (0.85–3.69)	0.125
	vs. T3	1.93 (1.67–2.23)	< 0.001	1.34 (1.15–1.57)	< 0.001	3.57 (1.87–6.82)	< 0.001	2.83 (1.47–5.44)	0.002	2.04 (1.05–3.93)	0.034	1.87 (0.96–3.65)	0.065
	vs. T4	2.09 (1.83–2.39)	< 0.001	1.40 (1.20–1.62)	< 0.001	5.10 (2.88–9.01)	< 0.001	4.24 (2.38–7.57)	< 0.001	2.76 (1.54–4.94)	< 0.001	2.74 (1.51–4.97)	< 0.001
pN stage (ref: N0)	vs. N1	1.55 (1.33–1.80)	< 0.001	NA		1.89 (0.96–3.71)	0.066	NA		1.29 (0.65–2.57)	0.471	NA	
	vs. N2	1.61 (1.36–1.90)	< 0.001	NA		2.06 (0.99–4.28)	0.054	NA		1.36 (0.64–2.87)	0.422	NA	
	vs. N3	1.77 (1.52–2.05)	< 0.001	NA		3.65 (2.11–6.33)	< 0.001	NA		2.29 (1.30–4.04)	0.004	NA	

*Uni* univariable; *Multi* multivariable; *OR* adjusted odds ratio; *CI* confidence interval; *ref* reference; *BMI* body mass index; *ASA* American Society of Anesthesiology; *Op* operation; *Lapa* laparoscopic; *Robot* robotic; *STG* subtotal gastrectomy; *TG* total gastrectomy; *PG* proximal gastrectomy; *NA* not applicable.

### FTR according to postoperative complication

**Figure 3:**
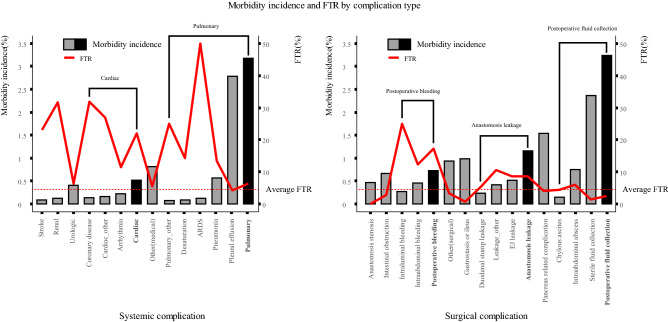
Incidence of morbidity and failure-to-rescue (FTR) rate according to complication types (the height of the gray or black box indicates the morbidity incidence and is displayed on the left y-axis. The red line indicates the FTR rate and is displayed on the right y-axis.)

Figure 3 shows the incidence and FTR by complication type after gastrectomy. Pleural effusion was the most common (incidence 2.78%) complication requiring a grade III or higher procedure or readmission, followed by sterile fluid collection and pancreas-related complications (incidence 2.36% and 1.53%, respectively). Ordered by FTR, acute respiratory distress syndrome (ARDS; FTR 50.00%), coronary artery disease (31.82%), renal complications (31.58%), and stroke (23.08%) were most often fatal once they occurred. Other surgical complications with higher FTR than the average (4.53%) were postoperative bleeding (17.24%), anastomosis leakage (8.65%), and intraabdominal abscess (5.88%). Patients with high-FTR index complications, such as ARDS, coronary artery disease, renal complications, stroke, and surgical complications, including postoperative bleeding or anastomosis leakage, were reviewed and their postoperative clinical courses were presented in a swimmer plot (Supplementary Fig. S1).

## Discussion

This study provided large-volume postoperative clinical data on adverse events (i.e., complications and mortality) in approximately 16,000 gastric cancer patients who underwent radical curative gastrectomy at a single referral center in South Korea, an East-Asian country with one of the highest incidences of stomach cancer in the world. The overall morbidity rate after radical gastrectomy was 10.49% when morbidity was defined as the occurrence of grade IIIa or higher complications according to the Clavien-Dindo classification or a readmission event. FTR after complications occurred in 4.53% of patients with morbidity, resulting in a 0.48% overall mortality rate from complications. We demonstrated that the risk factors of FTR include old age, high ASA score, and T stage, whereas sex, operation method, and resection extent were not associated with the FTR rate. Moreover, patients who underwent gastrectomy in 2016–2021 were less likely to die after morbidity than those who received surgery in 2006–2010.

Since its introduction in the early 1990s, FTR has been adopted as a quality indicator of mortality management^16,17^. With growing interest, more factors related to FTR were revealed, and the associations between various hospital systems and FTR have been investigated. Microsystem factors, such as nurse-to-patient ratio and intensive care unit (ICU) physician coverage^18–20^, and macrosystem factors, including hospital size and teaching status^11,21–25^, also affect the FTR rate. However, this study focused on investigating the predictive value of clinicopathologic and surgical factors associated with complications and FTR following gastrectomy in a specialty database constructed over 16 years in a high-volume center. We showed that the risk factors for morbidity and FTR were not identical and that the FTR risk factors for specific complications differed from those for all complications.

While conducting this FTR study, it was thought that the occurrence of complications and recovery from complications are distinct processes, and the risk factors associated with each of these processes could differ. The multivariable logistic regression models identified old age, high ASA score, and pathologic serosa exposure as common independent risk factors of morbidity, mortality, and FTR, which are consistent with previous studies^7,13,26–28^. On the other hand, male sex was a risk factor for morbidity and mortality but not for FTR. Compared with open surgery, minimally invasive surgery, including laparoscopic and robotic gastrectomy, might have lowered the morbidity rate but was not associated with a lower FTR rate once morbidity occurred. Similarly, total gastrectomy and proximal gastrectomy were not related to the FTR rate, though subtotal gastrectomy was associated with lower morbidity rates. Because the surgical factors of operation method and resection extent were only related to morbidity, they did not seem to affect how well the patients recovered from complications once morbidity developed.

To reduce post-surgery mortality, a mortality management approach should focus on reducing morbidity and saving patients from complications, thereby lowering the FTR rate. Morbidity reduction is, of course, the most crucial factor for patient safety and depends on the quality of the surgical team, whereas complication avoidance indicates the competency of the hospital, depending not only on the surgeon but also on the perioperative care team. This study shows that the stable performance of the surgical team impacts the morbidity rate and that the performance of the perioperative care team impacts the FTR rate.

The FTR rate of anastomosis leakage patients was lower in 2016–2021 compared with 2006–2010 (2.94% vs. 12.31%, *P* = 0.051), whereas the incidence of anastomosis leakage was not (1.32% vs. 1.16%, *P* = 0.510). In addition, the FTR risk factor analysis in the multivariable logistic regression model showed that patients who underwent gastrectomy in 2016–2021 were more likely to be rescued from complications than those who received surgery in 2006–2010 (OR 0.14, 95% CI 0.03–0.76, *P* = 0.023). In general, FTR rates are closely associated with hospital competency. Given the 16-year study period, the FTR rate may have been affected by changes in hospital characteristics and the development of non-operative care modalities. For example, the development of endoscopic intervention has enabled the consideration of endoscopic stent, negative pressure vacuum therapy, or endoscopic bleeder ligation as treatment options for anastomosis leakage or postoperative bleeding^29–34^. Moreover, the development of interventional radiology has led angiography to become one of the most important treatment modalities for managing patients with postoperative bleeding.

On the other hand, the macro- and microsystems associated with perioperative care also developed and changed from 2006 to 2021. Over the past few years, efforts have been made to improve the outcomes of hospitalized patients, including the onboarding of a surgical hospitalist, surgical ICU staff, and a rapid response team. The surgical hospitalist system, including a hospitalist with a surgical board, was established in 2016, representing the most significant change in perioperative care^35–37^. Although surgical staff members have the strength of knowing operative findings and the specific surgical risks of each patient, their busy schedules and prioritization of work performed in the operative theater mean they may fail to notice when a patient is off track. Early detection of and intervention for complications by a hospitalist could therefore be key to perioperative management. The surgical intensivist system, consisting of an ICU staff member with a surgical board, began in 2017 and may also have affected patients’ probability of survival with their knowledge of and experience with ventilator care and vital sign management. The surgical hospitalist’s and surgical intensivist’s close contact with the patients and knowledge of laboratory examination and medical treatment for surgical complications make these systems the most significant recent changes in perioperative care at our department level^38^. Changes in hospital-level also took place during the study period. A medical emergency system was established in 2019 whereby when a patient’s blood pressure or pulse worsens, a rapid response team is activated to check the patient’s situation and rapidly react^39^. Thus, even when the surgeon or physician is held in the operating room or during other procedures, the hospital’s system can respond immediately to take care of the patient in the ward or ICU to facilitate early detection and treatment of deteriorated patients.

In this study, the expected risk factors for high FTR were demonstrated. Regarding that some patients cannot recover from complications, a different clinical approach may be required in patients who are expected to have a high FTR rate. For example, endoscopic submucosal dissection(ESD) with more expanded criteria could be an alternative treatment option for octogenarian patients with severe morbidity. Additionally, downstaging of gastric cancer could be expected with neoadjuvant chemotherapy. Patients predicted to have a high FTR rate should be considered to receive surgical treatment cautiously.

To the best of our knowledge, this study is the most comprehensive investigation on gastric cancer patients’ FTR after gastrectomy in East Asia, which has the highest global incidence of gastric cancer. FTR has attracted attention as an essential indicator of mortality quality control, and our large-scale study of more than 15,000 people provides background information on how many complications and deaths have occurred after radical curative gastrectomy. Once complications occurred, the operation method and resection extent did not affect the FTR rate. Additionally, there is no evidence to suggest that FTR could vary according to the period after a major complication occurs following gastrectomy. However, our study demonstrated the possibility of a decrease in the FTR rate over periods.

This study has several limitations. First, this was a single-institution cohort-based study, so hospital characteristics were not investigated. A national or multicenter study with hospital characteristics could provide further information on comprehensive postoperative clinical outcomes for gastric cancer patients after gastrectomy. In addition, the risk factors of high FTR investigated in this study are challenging to correct in clinical practice. Exploring additional risk factors that can change perioperative management is essential. Next, we did not confirm which factors or developments decreased the FTR rate in recent years. Investigation of the diagnosis time of complication occurrence, treatment modality, and how the hospital system changed according to the year might reveal the reasons for the change in the FTR rate. Further study is needed to demonstrate whether changes in the hospital's macro-microsystems, as well as postoperative care, can be estimated as variables. It is necessary to further investigate how to save gastric cancer patients with complications.

This analysis of morbidity and mortality after gastric cancer surgery in a high-volume center demonstrated that male sex, high ASA score, and serosa exposure were risk factors for FTR. However, FTR differs by complication type, and the risk of FTR has decreased in recent years.

## Supplementary Information

Supplementary Information.

## Data Availability

The datasets used and analyzed during the current study available from the corresponding author on reasonable request.
