# Supplementary material for: Changes in failure to rescue after gastrectomy at a large-volume center with a 16-year experience in Korea
Source: Sci Rep. 2023 Mar 31;13:5252. doi: 10.1038/s41598-023-32593-6 (PMC10066195; doi:10.1038/s41598-023-32593-6)

**Supplementary Material**

**Supplementary Table 1.** Comparison of clinicopathologic features between patients with morbidity and those without

**Supplementary Table 2.** Comparison of clinicopathologic features between rescued patients and failure-to-rescue patients

**Supplementary Table 3.** Logistic regression models for risk factors of failure to rescue after gastrectomy in patients with anastomosis leakage

**Supplementary Fig. S1.** Swimmer plot showing the clinical course of patients with coronary artery disease, acute respiratory distress syndrome, renal complications, cerebrovascular disease, anastomosis leakage, and postoperative bleeding as an index complication

**Supplementary Table S1.** Comparison of clinicopathologic features between patients with morbidity and those without

|  | No. (%) |  |  |
| --- | --- | --- | --- |
| Characteristics | **Patients without morbidity**  **(n = 14308)** | **Patients with morbidity**  **(n = 1676)** | ***P* value** |
| Age, mean (SD), yr | 58.6 (12.0) | 62.6 (11.6) | <0.001 |
| <60 | 7287 (50.9) | 631 (37.7) | <0.001 |
| 60–79 | 6672 (46.6) | 951 (56.7) |  |
| ≥80 | 349 ( 2.5) | 94 ( 5.6) |  |
| Sex |  |  | <0.001 |
| Male | 8846 (61.8) | 1224 (73.0) |  |
| Female | 5462 (38.2) | 452 (27.0) |  |
| BMI,  mean (SD), kg/m2 |  |  |  |
| <18.5 | 574 ( 4.0) | 76 ( 4.5) | 0.057 |
| 18.5–23 | 5851 (40.9) | 628 (37.5) |  |
| 23–25 | 3768 (26.3) | 466 (27.8) |  |
| 25–30 | 3789 (26.5) | 458 (27.3) |  |
| ≥30 | 326 ( 2.3) | 48 ( 2.9) |  |
| ASA score |  |  | <0.001 |
| 1 | 5433 (38.0) | 510 (30.4) |  |
| 2 | 6699 (46.8) | 725 (43.3) |  |
| 3 | 2072 (14.5) | 403 (24.0) |  |
| 4 | 104 ( 0.7) | 38 ( 2.3) |  |
| Operation year |  |  | 0.714 |
| 2006–2010 | 4395 (30.7) | 531 (31.7) |  |
| 2011–2015 | 4662 (32.6) | 536 (32.0) |  |
| 2016–2021 | 5251 (36.7) | 609 (36.3) |  |
| Operation method |  |  | <0.001 |
| Open | 5220 (36.5) | 474 (28.3) |  |
| Laparoscopy | 6475 (45.2) | 1014 (60.5) |  |
| Robotic | 2613 (18.3) | 188 (11.2) |  |
| Resection |  |  | <0.001 |
| PG | 350 ( 2.4) | 44 ( 2.6) |  |
| STG | 11041 (77.2) | 1034 (61.7) |  |
| TG | 2917 (20.4) | 598 (35.7) |  |
| Dissection |  |  | <0.001 |
| D1+ | 7122 (49.8) | 624 (37.2) |  |
| D2 or more | 7186 (50.2) | 1052 (62.8) |  |
|  | **No. (%)** |  |  |
| Characteristics | **Patients without morbidity**  **(n = 14308)** | **Patients with morbidity**  **(n = 1676)** | ***P* value** |
| Operation time, mean(SD), minutes | 178.5 (58.9) | 196.1 (70.6) | <0.001 |
| pT |  |  | <0.001 |
| pT1 | 9098 (63.6) | 795 (47.4) |  |
| pT2 | 1520 (10.6) | 232 (13.9) |  |
| pT3 | 1733 (12.1) | 292 (17.4) |  |
| pT4 | 1957 (13.7) | 357 (21.3) |  |
| pN |  |  | <0.001 |
| pN0 | 10119 (70.7) | 998 (59.5) |  |
| pN1 | 1566 (11.0) | 239 (14.3) |  |
| pN2 | 1171 ( 8.2) | 186 (11.1) |  |
| pN3 | 1452 (10.1) | 253 (15.1) |  |
| AJCC 8^th^ stage |  |  | <0.001 |
| I | 9712 (67.9) | 898 (53.6) |  |
| II | 2154 (15.0) | 342 (20.4) |  |
| III | 2442 (17.1) | 436 (26.0) |  |

*SD* standard deviation; *BMI* body mass index; *ASA* American Society of Anesthesiology; *PG* proximal gastrectomy; *STG* subtotal gastrectomy; *TG* total gastrectomy; *AJCC* American Joint Committee on Cancer.

**Supplementary Table S2.** Comparison of clinicopathologic features between rescued patients and failure-to-rescue patients

|  | No. (%) |  |  |
| --- | --- | --- | --- |
| Characteristics | **Rescued patients**  **(n = 1600)** | **Failure to rescue patients**  **(n = 76)** | ***P* value** |
| Age, mean (SD), yr | 62.3 (11.6) | 69.1 (10.0) | <0.001 |
| <60 | 616 (38.5) | 15 (19.7) | <0.001 |
| 60–79 | 901 (56.3) | 50 (65.8) |  |
| ≥80 | 83 ( 5.2) | 11 (14.5) |  |
| Sex |  |  | 0.597 |
| Male | 1166 (72.9) | 58 (76.3) |  |
| Female | 434 (27.1) | 18 (23.7) |  |
| BMI,  mean (SD), kg/m2 |  |  |  |
| <18.5 | 68 ( 4.1) | 8 (10.5) | 0.019 |
| 18.5–23 | 595 (37.2) | 33 (43.4) |  |
| 23–25 | 449 (28.1) | 17 (22.4) |  |
| 25–30 | 444 (27.8) | 14 (18.4) |  |
| ≥30 | 44 ( 2.8) | 4 ( 5.3) |  |
| ASA score |  |  | 0.001 |
| 1 | 498 (31.1) | 12 (15.8) |  |
| 2 | 695 (43.5) | 30 (39.5) |  |
| 3 | 373 (23.3) | 30 (39.5) |  |
| 4 | 34 ( 2.1) | 4 ( 5.2) |  |
| Operation year |  |  | 0.321 |
| 2006–2010 | 502 (31.4) | 29 (38.2) |  |
| 2011–2015 | 511 (31.9) | 25 (32.9) |  |
| 2016–2021 | 587 (36.7) | 22 (28.9) |  |
| Operation method |  |  | 0.966 |
| Open | 453 (28.3) | 21 (27.6) |  |
| Laparoscopy | 967 (60.4) | 47 (61.9) |  |
| Robotic | 180 (11.3) | 8 (10.5) |  |
| Resection |  |  | 0.714 |
| PG | 43 ( 2.7) | 1 ( 1.3) |  |
| STG | 988 (61.7) | 46 (60.5) |  |
| TG | 569 (35.6) | 29 (38.2) |  |
| Dissection |  |  | 0.033 |
| D1+ | 605 (37.8) | 19 (25.0%) |  |
| D2 or more | 995 (62.2) | 57 (75.0%) |  |
|  | **No. (%)** |  |  |
| Characteristics | **Rescued patients**  **(n = 1600)** | **Failure to rescue patients**  **(n = 76)** | ***P* value** |
| Operation time, mean(SD), minutes | 195.7 (70.4) | 205.0 (74.4) | 0.263 |
| pT |  |  | 0.005 |
| pT1 | 773 (48.3) | 22 (28.9) |  |
| pT2 | 220 (13.8) | 12 (15.8) |  |
| pT3 | 276 (17.2) | 16 (21.1) |  |
| pT4 | 331 (20.7) | 26 (34.2) |  |
| pN |  |  | 0.034 |
| pN0 | 962 (60.1) | 36 (47.4) |  |
| pN1 | 228 (14.2) | 11 (14.5) |  |
| pN2 | 177 (11.1) | 9 (11.8) |  |
| pN3 | 233 (14.6) | 20 (26.3) |  |
| AJCC 8^th^ stage |  |  | 0.006 |
| I | 869 (54.3) | 29 (38.1) |  |
| II | 326 (20.4) | 16 (21.1) |  |
| III | 405 (25.3) | 31 (40.8) |  |

*SD* standard deviation; *BMI* body mass index; *ASA* American Society of Anesthesiology; *PG* proximal gastrectomy; *STG* subtotal gastrectomy; *TG* total gastrectomy; *AJCC* American Joint Committee on Cancer.

**Supplementary Table S3.** Logistic regression models for risk factors of failure to rescue after gastrectomy in patients with anastomosis leakage

|  |  | Uni |  | Multi |  |
| --- | --- | --- | --- | --- | --- |
| Characteristics | **Comparison** | **OR (95% CI)** | ***P*-value** | **OR (95% CI)** | ***P*-value** |
| Age, yr (ref : <60) | vs. 60–79 | 1.76(0.54–5.72) | 0.344 | NA |  |
|  | vs. ≥80 | 0(0–∞) | 0.992 | NA |  |
| Sex (ref: Female) | vs. Male | 3.24(0.41–25.46) | 0.264 | 4.84(0.56–42.00) | 0.153 |
| BMI, kg/m^2^  (ref: 18.5-22.9) | vs. <18.5 | 0(0–∞) | 0.990 | NA |  |
|  | vs. 23–24.9 | 0.59(0.17–2.05) | 0.408 | NA |  |
|  | vs. 25–29.9 | 0.26(0.05–1.27) | 0.095 | NA |  |
|  | vs. ≥30 | 0.74(0.08–6.56) | 0.787 | NA |  |
| ASA score  (ref: 1,2) | vs. 3, 4 | 0.39(0.09–1.80) | 0.388 | NA |  |
| Op year  (ref: 2006–2010) | vs. 2011–2015 | 0.93(0.30–2.87) | 0.899 | 0.63(0.18–2.17) | 0.461 |
|  | vs. 2016–2021 | 0.22(0.04–1.06) | 0.059 | 0.14(0.03–0.76) | 0.023 |
| Op method (ref: Open) | vs. Lapa | 0.68(0.20–2.29) | 0.536s | NA |  |
|  | vs. Robot | 0.56(0.12–2.70) | 0.472 | NA |  |
| Resection (ref: STG) | vs. TG | 0.81(0.29–2.28) | 0.687 | NA |  |
|  | vs. PG | 0(0–∞) | 0.989 | NA |  |
| Dissection (ref: D1+) | D1+ vs. D2 | 10.61(1.37–82.16) | 0.024 | 20.48(2.3–182.37) | 0.007 |
| pT stage  (ref: T1) | vs. T2 | 0(0–∞) | 0.990 | 0(0–∞) | 0.992 |
|  | vs. T3 | 1.42(0.39–5.19) | 0.597 | 0.59(0.14–2.50) | 0.473 |
|  | vs. T4 | 1.67(0.49–5.63) | 0.411 | 0.48(0.12–1.92) | 0.302 |
| pN stage  (ref: N0) | vs. N1 | 2.19(0.61–7.86) | 0.231 | NA |  |
|  | vs. N2 | 2.62(0.63–11.00 | 0.187 | NA |  |
|  | vs. N3 | 0.52(0.06–4.39) | 0.552 | NA |  |

Uni univariable; Multi multivariable; OR adjusted odds ratio; CI confidence interval; ref reference; BMI body mass index; ASA American Society of Anesthesiology; Op operation; Lapa laparoscopic; Robot robotic; STG subtotal gastrectomy; TG total gastrectomy; PG proximal gastrectomy; NA not applicable

**Supplementary Fig. S1.** Swimmer plot showing the clinical course of patients with coronary artery disease, acute respiratory distress syndrome, renal complications, cerebrovascular disease, anastomosis leakage, and postoperative bleeding as an index complication


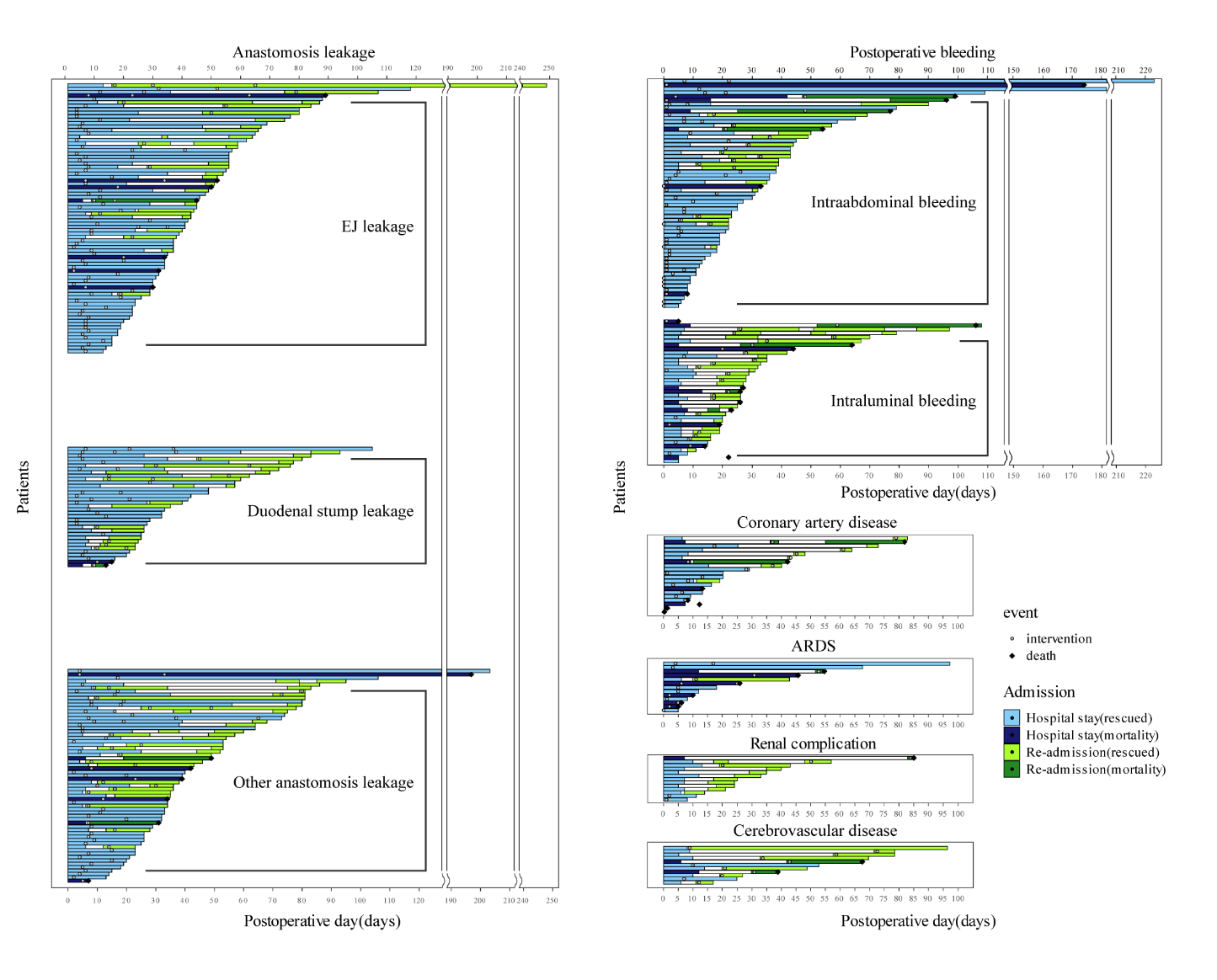

Supplement: Supplementary file 1 — Supplementary Information. [file 41598_2023_32593_MOESM1_ESM.docx]
